# Investigating Climate Change and Reproduction: Experimental Tools from Evolutionary Biology

**DOI:** 10.3390/biology1020411

**Published:** 2012-09-13

**Authors:** Vera M. Grazer, Oliver Y. Martin

**Affiliations:** ETH Zurich, Experimental Ecology, Institute for Integrative Biology, Universitätsstrasse 16, CH-8092 Zurich, Switzerland

**Keywords:** experimental evolution, sexual selection, global warming, speciation, extinction

## Abstract

It is now generally acknowledged that climate change has wide-ranging biological consequences, potentially leading to impacts on biodiversity. Environmental factors can have diverse and often strong effects on reproduction, with obvious ramifications for population fitness. Nevertheless, reproductive traits are often neglected in conservation considerations. Focusing on animals, recent progress in sexual selection and sexual conflict research suggests that reproductive costs may pose an underestimated hurdle during rapid climate change, potentially lowering adaptive potential and increasing extinction risk of certain populations. Nevertheless, regime shifts may have both negative and positive effects on reproduction, so it is important to acquire detailed experimental data. We hence present an overview of the literature reporting short-term reproductive consequences of exposure to different environmental factors. From the enormous diversity of findings, we conclude that climate change research could benefit greatly from more coordinated efforts incorporating evolutionary approaches in order to obtain cross-comparable data on how individual and population reproductive fitness respond in the long term. Therefore, we propose ideas and methods concerning future efforts dealing with reproductive consequences of climate change, in particular by highlighting the advantages of multi-generational experimental evolution experiments.

## 1. Introduction

Today, natural as well as anthropogenic climate change and associated negative impacts on biodiversity are widely acknowledged [1]. Many reviews on climate change have thus summarized the wide-ranging biological changes that can be observed in nature [2,3,4,5,6,7,8,9]. For example, it is clear that global warming has begun to affect many species, changing fundamental characteristics, such as body size [10] or phenology [11,12,13,14,15]. The multiple consequences of climate change are still under intense scrutiny, especially as models estimating global biodiversity gains and losses could still be improved. Potential climate change impacts are being modeled with increasing realism as species distribution prediction techniques become more sophisticated [16]. However, in order to achieve greater accuracy, such models will increasingly require high quality and biologically relevant input data. Compared to the rapid advances in climate change ecology and environmental sciences, which provide abiotic information about climate change, evolutionary biology, which could provide some of the necessary biotic information in this particular context, seems to be lagging behind. Therefore, we advocate for coordinated advances across both fields. On this note, after briefly reviewing some aspects of current evolutionary research on environmental change and identifying certain caveats, we will outline promising experimental avenues for future efforts.

## 2. Single Generation Studies on Traits Associated with Reproduction

Taking the evolutionary perspective, climate change is a two-edged sword. On the one hand, climate change can increase extinction risk of populations if migration to different habitats is not possible or the adaptive potential in the original changing habitat is low. In particular, the combination of climate change and habitat fragmentation could critically reduce local population sizes [17,18,19], which reduces the chances of persistence due to loss of genetic diversity [20,21] or stochastic demography [22,23]. On the other hand, climate change may also promote adaptation [5,7,24,25] or even speciation [26,27,28] by opening up novel niches and stimulating on-going genetic change. However, adaptation crucially depends on how rapidly conditions change [29]. Furthermore, the role of microevolutionary changes in response to climate change remains largely unclear, in particular, because phenotypic adaptations could either delay or facilitate genetic change [30,31,32,33]. 

A common and very important feature of extinction, adaptation and speciation is that these processes are governed by the longevity and reproduction of individuals in populations affected by climate change. Specifically, successful reproduction allows populations to grow, which minimizes extinction risk and increases the potential for adaptation or divergence and speciation. In contrast, slow or even negative population growth due to unsuccessful reproduction and potentially reduced survival can decrease population size, rendering populations more vulnerable to extinction. Therefore, measuring longevity and reproductive success can provide important evolutionary information, which may potentially be crucial for understanding the short and long-term impacts of climate change [34].

An essential question, therefore, is to ask what evidence we have that climate change is already affecting reproduction. Clearly, reproduction is a multifaceted process, and is based on many traits and underlying genes. Nevertheless, with regard to the environmental dependence of many reproductive traits, we can draw on a large body of ecological work (Table 1 and Table 2). For example, many arthropods have been used in multiple-exposure studies, where individuals are subjected to different abiotic conditions and reproductive traits measured (see examples in Table 2). So far, multiple-exposure studies have typically reported the short-term responses of reproductive traits to discrete intervals of an abiotic factor such as temperature. Commonly in this kind of experimental set-up, the measure of interest is how many eggs or offspring are produced under the given conditions. Based on these experiments, we draw the following conclusions, which we think are especially important when investigating impacts of climate change on reproduction:

First, many multiple-exposure experiments report a very narrow parameter window, such as an optimal temperature, where shifts of only, for example, +2° or –2 °C lead to pronounced deterioration in reproductive output. In a natural setting, this may suggest that only slight climate changes, for example in terms of decreased minimum or increased maximum local temperatures or altered environmental cues, might profoundly affect total population fitness.

Second, multiple-exposure experiments usually analyze reproductive traits in terms of the rate of natural population increase, thus allowing comparisons across studies. Along the same lines, it would be highly beneficial if future studies on climate change were based on the same measurement and analysis of reproductive fitness. Importantly, a standardized method of assessing reproductive output of a population following an environmental change would allow models to incorporate an important facet of adaptive potential based on real data.

Third, up to now, multiple-exposure studies have generally employed a single generation experimental approach. Specifically, the parental generation is exposed to different conditions and then reproductive fitness is measured in terms of F1 output. However, with regard to providing evolutionarily meaningful data for climate change research, it would be necessary to perform analogous experiments over multiple generations. In particular, single generation studies often lack information concerning longevity of the parental and filial generations, and hence only provide a snapshot of the reproductive phase of an organism.

Fourth, depending on the organism, different reproductive traits are usually measured to assess fitness. For example, in arthropods, the rate of natural increase (see 2^nd^ point) is often based on fecundity (see examples in Table 2) although this omits information concerning how many offspring actually survive to maturity. Therefore, not all reproduction-related traits are equally useful. Overall, the most relevant measurement, as it translates to population productivity, is probably lifetime reproductive success. 

In summary, the rich diversity of examples in Table 1 and Table 2 provides evidence that many reproductive traits are strongly affected by environmental factors. Furthermore, these findings illustrate that environmental change can have positive or negative effects on reproduction, suggesting that experimental data might be necessary in order to predict consequences of climate change more precisely. In the following, we will take up and elaborate on some of these aspects to provide ideas on how future research could experimentally address impacts of climate change on reproduction. Specifically, we give arguments for using lifetime reproductive success or population growth as common measures to assess fitness. Thereafter, we present experimental evolution as a powerful tool to achieve and implement the ideas proposed. Finally, we draw conclusions and suggest directions for streamlining evolutionary research on reproduction in response to climate change.

**Table 1:** Examples of studies assessing consequences of climate change, in particular effects of temperature, on reproductive traits. The studies are divided between lab-based experiments using different controlled temperature treatments, and experiments investigating reproductive traits in the field by observing or simulating changes to natural and anthropogenic climate change.

Environment	Species	Environmental factor under investigation ^1,2^	Outcome on reproductive traits ^2^	Reference
***Lab environment***	Wolf spider	Temperature	Warmer temps gradually decreased courtship effort and copulation duration	[35]
	*Pardosa astrigera*	[16,20,**24**,28,32 °C]		
	Dung fly	Temperature	Warmer ambient temps gradually decreased copulation duration	[36]
	*Sepsis cynipsea*	[17,18,20,24,26,29 °C]		
	Stingless wasp	Temperature	At warmer temps fewer primary spermatocytes	[37]
	*Trichogramma brassicae*	[**23**,35,44 °C]		
	Adzuki bean beetle	Temperature	Warmer temps reduced mating duration and number of sperm transferred	[38]
	*Callosobruchus chinensis*	[17,25,33 °C]		
	Argentine ant	Temperature	Warmer temps decreased development time up to 30° (60d *vs*. 160d at 21°)	[39]
	*Linepithema humile*	[18,21,24,26,**28**,30,32 °C]		
	Fruit fly	Temperature	At low temp. female-biased offspring sex ratio	[40]
	*Drosophila melanogaster*	[low 5.5–14.5 °C, constant 25°C, high 20–33.5 °C]		
	Cricket	Temperature	Different aspects in male mating call increased or decreased due to temp	[41]
	*Allonemobius socius*	[24,31 °C]		
	Great tit	Temperature	In 5 of 6 experiments (1999–2004) birds from the warm treatment nested earlier	[42]
	*Parus major*	Warm: summer temps of 1998, cold: summer temps of 1986		
	Common starling	Temperature	Timing of testicular maturation initiated by photoperiod not temp.	[43]
	*Sturnus vulgaris*	[20 °C *vs*. 5 °C;18 *vs*. 8 °C]		
	Fathead minnow	Temperature	Increased temp. leads to higher sensitivity of vitellogenin expression	[44]
	*Pinnephales promelas*	[20,**25**,30 °C]		
	Leopard gecko	Temperature	In colder temp. more androgen receptors expressed in testes	[45]
	*Eublepharis macularius*	[29,18 °C]		
	Veiled chameleon	Temperature	Longer development time and higher egg mortality at higher temps	[46]
	*Chamaeleo calyptratus*	[25,28,30 °C]		
***Natural environment***	Fall webworm	Increased annual temps 1975–2002	Shift from bivoltinism to trivoltinism	[47]
	*Hyphantria cunea*			
	Dragonfly	Natural temp. variation plus artificial warming	Not faster than univoltine development	[48]
	*Orthetrum cancellatum*	[ambient, +2, +4, +6 °C]		
	Kentish plover	Natural temp. variation 2005–2006	Increased biparental nest attendance during temp. peaks	[49]
	*Charadrius alexandrinus*			
	Butterflies & moths	Increased annual mean summer temps1864–2008	Increased voltinism a general trend	[50]
	1117 *sp.*			
	Spruce bark beetle	Climate change modeling (3 SRES scenarios (IPCC)) 1961–2100	Predicted shift to bivoltinism in 50% of years by 2050 if temps increased by +2.4–3.8 °C 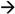 increased pest	[51]
	*Ips typographus*			
	Collared flycatcher	Natural variation during years 2003, 2005–2007	Maternal yolk hormone (androstenedione) transfer highly sensitive e.g., via body condition	[52]
	*Ficedula albicollis*			
	Greater snow goose	Temp., precipitation and snow cover variation 1994–2004	Warm spring temps and low snow cover: denser & earlier nesting, but reduced size and mass of fledglings causing decrease in RS	[53]
	*Chen caerulescens*			
	Barn swallow	Spring and summer temperatures 2000–2002	Egg mass increased with the temp. 2–5d before laying; temp. effect on carotenoid and immune factors deposition in eggs	[54]
	*Hirundo rustica*			
	Tuatara	Model with geographical, microclimatic and biophysical data until 2080	All male clutches predicted without adaptations; behavioral nesting adjustment unlikely	[55]
	*Sphenodon guntheri*			
	Lizard	Clinal gradient (19° lat.) in East Australia 2003–2004	Nest relocation to different sites to normalize nest temps and assure equal sex-ratio	[56]
	*Physignathus lesueurii*			
	Grey seals	Total rainfall in Octobers 1996–2004	Strong sexual selection in wet years, whereas more males reproduce in dry years	[57]
	*Halichoerus grypus*			
	African buffalo	Natural variation in precipitation 1978–1998	Wet 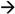 male-biased sex ratio, dry 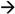 female-biased sex ratio, indicating condition-dependent sex-ratio distorter genes	[58]
	*Syncerus caffer*			
	Leatherback turtle	Natural variation in precipitation 1987–2003	Increased precipitation has cooling effect on nests, leading to more males	[59]
	*Dermochelys coriacea*			

^1^ See used temperature treatments in square brackets. Temperatures are given in degrees Celsius. The number in bold indicates the standard rearing temperature. Cf. references for further details;^ 2^ The abbreviations temp./temps are used for temperature(s) and RS for reproductive success (number of offspring).

**Table 2:** Examples of studies assessing effects of climate change on reproductive success (RS) including fecundity, fertility or number of offspring. The studies are divided between lab-based experiments using different controlled temperature treatments, and experiments investigating RS in the field by observing or simulating changes to natural and anthropogenic climate change.

Environment	Species	Environmental factor under investigation ^1,2^	Outcome on mean RS ^2^	Reference
***Lab environment***	Serpentine leafminer	Warmer temps	**Increase** until T_opt_ (=30° with fecundity 406 eggs)	[60]
	*Liriomyza trifolii*	[15,20,25,30,35]		
	Olive fruit fly	Warmer temps	**Decreased** production of mature eggs in elevated temps *vs.* control	[61]
	*Bactrocera oleae*	[18.3–23.9(control), 18.3–35.0, 18.3–37.8]		
	Fruit fly	Warmer temp.	**Increased** fec. overall via mother, slight decreasing effect via father	[62]
	*Drosophila melanogaster*	[18,25]		
	Fruit fly	Warmer or colder temp.	**In-/decreased** RS (84 at 29°, 79 at 25°, 38 at 18°)	[63]
	*Drosophila melanogaster*	[18,**25**, 29] for 1 day		
	Fruit fly	Cold exposures	**Decreased** number of offspring in multiply and sustained cold exposed flies	[64]
	*Drosophila melanogaster*	[constant 22°, 10h at –0.5°, multiple times 2h at –0.5°, 2h at –0.5°]		
	House fly	Warmer temps	**Increase** until T_opt_ (=30° with fecundity 495 *vs*. 118 and 433 at 20°and 25°)	[65]
	*Musca domestica*	[20,25,30,35]		
	Goldenrod gall fly	Warm spring temp	**Decreased** fec. if unfrozen (199 eggs at 12° *vs*. 256 at 0°)	[66]
	*Eurosta solidaginis*	[0,12] after overwintering		
	Hymenopteran parasitoid	Warmer temps	**Increased** lifetime fec. (62 eggs at 30° *vs*. 45–59 at other temps)	[67]
	*Trichogramma buesi*	[12,15,**20**,25,30,35]		
	Hymenopteran parasites	Warmer temp.	**Decreased** fec. in all three species (e.g., in *P. palitans* 76 *vs*. 579 eggs)	[68]
	*Praon palitans, Trioxys utilis, Aphelinus semiflavus*	[27,**21**]		
		Colder temp.	**Decreased** fec. in all three species	
		[16,**21**]		
	Hymenopteran parasitoids	Warmer temp.	**Increased** offspring number in all 5 species (e.g., in *S. cameroni* 6 offspring at 21 *vs*. 12 at 29°)	[69]
	*Muscidifurax raptor, M. zaraptor, M. uniraptor, Spalangia cameroni, S. endius*	[**21**,29]		
	Hymenopteran parasite	Warmer temps	**Increase** until T_opt_ (=22.5° with 163 offspring, 112 at 25°, 80 at 27.5° and higher)	[70]
	*Trissolcus oenone*	[15,17.5,20,22.5,**25**, 27.5,30,32.5,35]		
	Silverleaf whitefly	Warmer temps	**Decrease** (324 eggs/female at 20° *vs*. 22 eggs at 35°)	[71]
	*Bemisia argentifolii*	[15,20,25,27,30,35]		
	Butterflies *: Hipparchia semele,*	Warmer temp.	**Decreased** fec. in *P. aegeria*, fec. highest at 30° in other 3 species	[72]
	*Coenonympha pamphilus, Aphantopus hyperantus, Pararge aegeria*	[30,**25**]		
		Colder temp. [20,**25**]	**Decreased** fec. in all 4 sp.	
	Butterfly	Colder temp.	**Decreased** lifetime fec. (85 *vs*. 133)	[73]
	*Pararge aegeria*	[19,27]		
	Spruce bud moth	Warm/cold temps	**Decreased** fec. at extremes (59 viable eggs at 10°, 53 at 25° *vs*. 84 and 86 eggs at 15 and 20°)	[74]
	*Zeiraphera canadensis*	[10,15,**20**,25]		
		Fluctuating temp.		
		[alternating between 10 and 25]	**Decreased** fec. (51 viable eggs, see above)	
	Lesser peach tree borer	Warmer temps	**Increased** fec. until T_opt_ (=30°; 221 eggs *vs*. 0–122 at lower temps)	[75]
	*Synanthedon pictipes*	[15.2,20.8,23.5,**26.9**, 30.3,33.6,37.8]		
	Spruce bark beetle	Warmer temps.	**Increased** fec. until T_opt_ (=30° with 24 eggs, 10 eggs at 15°)	[76]
	*Ips typographus*	[12,15,20,25,30,33]		
	Mexican bean beetle	Warmer temp.	**Decreased** fecundity	[77]
	*Epilachna varivestis*	[27,**22**]	(6 eggs *vs*. 75)	
		Colder temp.	**Decreased** fecundity	
		[17,**22**]	(38 eggs *vs*. 75)	
	Stored product pest beetles	Sinusoidal fluctuation	*T.c*.: **increased** fec. in fluctuating *vs*. constant regime	[78]
	*Tribolium castaneum, Trogoderma*	[10° range, mean 25]		
	*inclusum, Sitophilus oryzae*	*vs*. constant 25]	*T.i., S.o*.: **no effect**	
	Stored product pest beetle	Warmer temp.	**Increased** RS of polyandrous beetles at warmer temp.	[79]
	*Tribolium castaneum*	[**30**,34]		
	Stored product pest beetles	Warmer temps	**Increased** fec.: *T. cast.*: 19, 51 and 57 eggs, *T. conf.*: 15, 38 and 43 eggs	[80]
	*Tribolium castaneum, T. confusum*	[24,29,34]		
	Wolf spider	Cold/warm temps	**Increase** until T_opt_ (=24° with lifetime fec. of 67 *vs*. 16–66 at other temps)	[81]
	*Pardosa astrigera*	[16,20,**24**,28,32]		
	Cotton aphid	Warmer temps	**Increased** no. of offspring until T_opt_ (=30° with 3.1/day, 1.8/day at 15°, 0 at 35°, 3.1/day with 25/30° and 2.3/day with 30/35°)	[82]
	*Aphis gossypii*	[15,20,**25**,30,35]		
		Fluctuating temp.		
		[25/30,30/35]		
	Soybean aphid	Warmer temp. [30,**25**]	**Decrease** (23 *vs*. 73 eggs)	[83]
	*Aphis glycines*	Colder temp. [20,**25**]	**Stable** (75 *vs*. 73 eggs)	
	Corn aphids: *Rhopalosiphum padi, Sitobion avenae, Metopolophium dirhodum*	Warmer temps	*R.p.*: **Increase** (T_opt _= 27.5°), *S.a.*: **Decrease** (T_opt _= 18°), *M.d.*: **Decrease** (T_opt _= 18°)	[84]
		[18,22,25,27.5,30]		
	Wheat aphid	Warmer temps	**Decreased** offspring number from 35 at 15° to 34 (20°), 17 (25°) and 5 (30°)	[85]
	*Diuraphis noxia*	[5,10,15,20,25,30]		
	Western tarnished plant bug	Warmer temps [12.8,15.6,21.1,26.7, 32.2,35.0,37.8]	**Increase** until T_opt_ (=26.7°, 178 eggs *vs*. 38–140 at other temps)	[86]
	*Lygus hesperus*			
	Predatory mites	Warmer temp.	**Increased** egg laying rate in all 4 species	[87]
	*Galendromus longipilis, Neoseiulus fallacis, Phytoseiulus macropilis, Proprioseiopsis temperellus*	[13.3,26.4]		
	Predatory mite	Warmer temps	**Increase** to T_opt_ (=25°; 34 *vs*. 17–30 eggs at other temps)	[88]
	*Amblyseius largoensis*	[15,20,**25**,30,35]		
	Citrus rust mite	Warmer temps	**Increased** fec. from 2 eggs at 14° up to 15 at 27°, then decrease	[89]
	*Phyllocoptruta oleivora*	[12,14,17,19,21,23,25,27,29,31,33]		
	Predatory mite	Warmer/colder temps	**Decreased** fec. 8 and 16 eggs at 20 and 30° *vs*. 18 at 25°	[90]
	*Amblyseius californicus*	[20,**25**,30]		
	Predatory mite	Warmer temp. [30,**25**]	**Decrease** (31 *vs*. 57 eggs)	[91]
	*Hypoaspis miles*	Colder temps [15,20,**25**]	**Decrease** (33, 49, 57 eggs)	
	Predatory mite	Warmer temps	**Decreas**e (61 eggs at 21° *vs*. 53 and 26 at higher temps)	[92]
	A *mblyseius fallcis*	[21,27,32]		
	Water flea	Warmer temps	**Decreased** brood size if warmer than T_opt_ (=15°)	[93]
	*Daphnia parvula*	[5,10,15,20,25,30]		
	Water fleas	Warmer temps	**Increase** until T_opt_^2 ^= 20° for both species (*D. pulex* 56 offspring; *D. magna* 66)	[94]
	*Daphnia pulex, Daphnia magna*	[15,20,25,30]		
	Copepod	Warmer temps	Increased fec. until T_opt_^2^ = 17° then decrease	[95]
	*Acartia clausi*	[2.5,6.9,10.0,12.6,14.7, 17.0,19.4,21.7,25.0]		
	Reef damselfish	Warmer temps	**Decreased** clutch size and egg area at elevated temps *vs*. 28.5°	[96]
	*Acanthochromis polyacanthus*	[**28.5**,30,31.5]		
	Whitefish	Warmer temps	**Decreased** RS (more unfertilized and abnormal eggs at temps above 7°)	[97]
	*Coregonus lavaretus*	[4–5, 7–8, 9–10, 11–12, 13–14]		
	Palmate newts	Warmer temps	**Decreased** fec. (ca. 35 eggs at 22° *vs*. ca. 80 at 14 or 18°)	[98]
	*Lissotriton helveticus*	[14,18,22]		
	Grass lizard	Warm/cold temps	**Decreased** number of offspring per year (10 at 24°, 15 at 28°, 9 at 32°)	[99]
	*Takydromus septentrionalis*	[24,**28**,32]		
	Three-lined skink	Cold and hot treatments with contrasting duration of cage heating	**Increased** number of eggs per clutch (6.9 in hot *vs*. 6.3 in cold treatment)	[100]
	*Bassiana duperreyi*			
***Natural environment***	Butterfly	Warmer mean temps [23.1,25.1,29.3]	**Increase** (egg laying rate increases with temp.: 9.2, 11.3, 18.0 eggs per day)	[101]
	*Speyeria mormonia*			
	Warbler	Minimum, mean and max. temps 1973–2002	**Increased** clutch sizes over study period	[102]
	*Acrocephalus sp.*			
	Pied flycatcher	1943–2003: climatic factors at wintering ground in Africa	Highly variable RS	[103]
	*Ficedula hypoleuca*			
	Pied flycatcher	Fluctuation, warming	**Decreased** clutch size (birds breeding earlier and laying fewer eggs, most likely caused by increased spring temps)	[104]
	*Ficedula hypoleuca*	data for 2 to 11 years from each of 80 study areas in Europe		
	Coast nesting birds	High tide fluctuation (max. high tide increased twice as fast as mean high tide over the 4 decades, causing greater risk of flooding of nests)	**Decreased RS** (based on reproductive data from 26 years)	[105]
	*Haematopus ostralegus*			
	Seabirds	1996–1999 warmer sea surface temps between –0.5 and 0.4°	**Decreased RS** (potentially due to lower food abundance)	[106]
	*Uria aalge, Rissa tridactyla*			
	Common buzzard	Higher summer precipitation	**Decreased** lifetime RS	[107]
	*Buteo buteo*	Warmer (1989–2000)	**Increased** lifetime RS	
	Bivalve	Warmer water temps 1969–2007	**Decreased** population size (recruitment)	[108]
	*Macoma balthica*			
	Common frog	Global warming + 1.02º 1983–2006 (incl. heat wave 2003)	**Decrease** (2004 lowest fec. in the dataset, 2003 event more damaging than long term temp. increase)	[109]
	*Rana temporaria*			
	Chinese alligator	Increase in March/ April temps between 1987–2005	**Increased** clutch size with increasing temp.	[110]
	*Alligator sinensis*			
	Red squirrel	Natural environmental variability 1989–1998	Early breeding females with intermediate RS favored by selection	[111]
	*Tamiasciurus hudsonicus*			
	African lion	Warmer temperatures in Tanzania 1964–2001	More abnormal sperm in males with dark manes, **potential for decrease** in RS with climate change	[112]
	*Panthera leo*			
	Human	Global air temperatures from 1900–1994	**Decrease** in yearly birth rates	[113]
	*Homo sapiens*			

^1^ See tested temperatures in square brackets. Temperatures used are in degrees Celsius. The number in bold shows the standard rearing temperature. Cf. references for further details on experimental methods;^ 2^ Abbreviations: T_opt_ = optimal temperature where RS (fecundity, fertility or offspring) was maximized, temp./temps = temperature(s) and fec. = fecundity.

## 3. Measuring Reproduction

Reproduction involves multiple traits, which may be measured to infer fitness of an individual. The most widely used fitness measure is probably fecundity, which is the number of eggs an individual produces. However, depending on the organism, fecundity is usually assessed in different ways. Specifically, animal studies have used, for example, total number of eggs, total number of clutches, number of clutches per breeding season (e.g., voltinism) or single clutch size (see examples in Table 2). Therefore, comparing fecundity among species and among studies is often difficult. Furthermore, fecundity in terms of eggs and brood sizes etc. potentially depends to a very large part on the female. In particular, a female’s available energy and general effects due to body size pose large constraints for fecundity. Without correcting for these effects, comparisons among individuals might be biased. Furthermore, different individuals may pursue different life-history strategies, such as via a trade-off between number and size of eggs [114]. In addition, sexual conflict research suggests that males can enhance females’ fecundity via gonadotropic substances [115] and the outcome of this manipulation could be different for each male-female combination [116,117]. Therefore, in particular with promiscuous organisms, it might not be sufficient to only measure fecundity at a single time point and under (often unnatural) monogamous conditions. 

Because of these caveats and with regard to climate change research, it might be necessary to take a step away from individual fitness towards measuring population fitness. Alternatively, if focusing on the individual level, future studies should account for multiple traits that contribute to total reproduction, such as development time, egg hatching success, fertility, (re-)mating success or survival. Ideally, the use of a single fitness measure, which incorporates multiple traits, would allow improved comparisons across different studies and species. For this purpose, we propose using a single currency such as lifetime reproductive success, because it includes information about fecundity and survival of the mother and development and survival of the offspring. Ultimately, lifetime reproductive success could be used to estimate population growth, which is a key factor determining population resilience, for example, in population viability analysis [118,119]. In combination with estimates regarding population sizes, it might be possible to improve predictions of climate change impacts [119]. Lab and manipulated field experiments could thereby provide standardized information concerning how lifetime reproductive success, and hence population growth, are affected by environmental change. How future studies could approach this experimentally is discussed in more detail in section 5.

Depending on the organism, however, it may simply not be possible to assess lifetime reproductive success. In this case, it may be crucial to measure a suite of reproductive traits and longevity if possible. Multiple measurements of reproductive success over time to estimate reproductive rate, in combination with information about average life span, could allow inferences concerning population growth. 

## 4. Sexual Selection and Climate Change

Many pre- and postcopulatory reproductive traits can be affected by both natural and sexual selection [120]. This may include morphological, physiological, behavioral or immunological traits, which are used, for example, for mate choice or competition. There is, therefore, considerable potential for positive or negative interactions between natural and sexual selection with consequences for reproduction [121]. Individuals might fail to produce offspring if they are unable to compete in pre- or postcopulatory sexual selection, despite possessing optimal traits for survival in a current or changing environment. In that sense, sexual selection could oppose the direction of natural selection [121]. In contrast, as good genes models of sexual selection predict, if sexually selected traits are honestly linked with fitness, sexual selection might also reinforce natural selection [121]. In general, it is conceivable that such effects might translate from the individual to the population level, thereby affecting population productivity and hence viability. 

With regard to climate change, natural selection can advance adaptation to novel conditions and this crucially determines persistence of local populations [31]. So far, factors such as genetic diversity, heritability or phenotypic plasticity have been argued to contribute to population adaptability [30,31,122]. However, beyond a few experimental evolution studies investigating the interplay of natural and sexual selection [123,124,125], there is a clear lack of knowledge about the role of sexual selection for adaptation in climate change research. In particular, evolutionary studies generally have not implemented variable environments, but instead employed common garden conditions. In future studies, it would be important to incorporate fluctuating and temporally changing environments to attain greater environmental reality and investigate sexual selection and adaptation in response to more realistic scenarios. Furthermore, as a next step, lab-based findings could be replicated in more natural settings, in order to verify how robust the results are, with respect to the environmental context.

Based on natural and sexual selection pressures, climate change may result in different evolutionary outcomes. Beside adaptation for persistence *per se*, which is reviewed elsewhere, for example, in Candolin and Heuschele [121], we will briefly discuss reproductive isolation and extinction, focusing on the potential role of sexual selection.

### 4.1. Reproductive Isolation

Biodiversity depends on the coexistence of species, which are reproductively isolated. However, the degree of isolation varies along a continuum. In nature, therefore, species boundaries can become stronger or weaker via natural and sexual selection. In this context, climate change could have an important role. For example, climate change creates fluctuating conditions in space and time and can generate variability among individuals in a population. This might increase the potential for adaptive divergence and divergent sexual selection. Similarly, among different populations, adaptation following climate change could progress along different routes and isolation may be facilitated by sexual selection. Thereby, assortative mating and mate discrimination against incoming migrants or hybrids could act as diverging agents. In general, famous examples of fish and insect radiations suggest that sexual selection might play a role for reproductive isolation to arise [126]. Nevertheless, there is a clear lack of targeted experiments investigating under which exact circumstances sexual selection contributes to reproductive isolation.

In contrast, climate change may reduce the size of local populations, such that the density of individuals becomes lower than that favored by sexual selection. In particular, promiscuous species may not get access to enough mates. Presumably, species boundaries could weaken if heterotypic crosses become more frequent, for example, if isolation is mainly behavioral. Future studies could investigate this and similar hypotheses, which may provide important insights regarding impacts of climate change on mating systems and hence biodiversity.

### 4.2. Extinction

Over time, reproductive traits are shaped by natural and sexual selection within the environmental context in order to achieve high fitness. Theoretically, a population may reside on a peak in the fitness landscape. However, climate change can progress very rapidly, not only changing long term means, but also the frequency and strength of extreme weather events [127]. Therefore, environmental conditions may increasingly change dramatically within a few generations. With regard to sexually selected traits, which are not only optimized in terms of the environment, but also across the sexes, this may have unexpected consequences. Tanaka [128] formulated a model addressing this problem, which specifically investigated a system with a preference in one sex and a correlated costly ornament in the other sex in a system subjected to rapid environmental change. Results are formulated in terms of total selection load, which is the sum of all costs for maintaining the preference and the ornament. Most importantly, he shows that after the rapid change, there is a burst of selection load, because the expression of the costly ornament is not adapted. Additionally, the preference is initially mismatched and can only track the novel optimum of the ornament slowly. Ultimately, this burst of selection load may increase extinction risk.

Tanaka’s model [128] has very clear implications for biodiversity research, because the investigated mechanism potentially holds true for many genetically correlated traits, which collectively contribute to the selection load in a population. In particular, traits involved in sexual conflict, which have been shown to cause very high costs in both sexes, could fatally exacerbate extinction risk.

Regarding current species at risk, there is controversial evidence from comparative studies indicating a potential negative effect of sexual selection, for example, in birds [129,130,131] but not in mammals [132]. In contrast, lab experiments suggest that sexual selection might also act beneficially against extinction, such as by selecting against selfish genetic elements [133] or ameliorating inbreeding depression [134,135]. Clearly, more research efforts are urgently needed to get a better understanding of the circumstances and precise mechanisms of sexual selection, leading to increased or decreased extinction risk.

## 5. Experimental Evolution Simulating Climate Change Impacts

In order to experimentally investigate evolutionary changes due to climate change systematically, it will be necessary to manipulate evolutionary drivers and mechanisms in detail and to implement increasing levels of ecological reality. In many contexts, experimental evolution enables researchers to achieve these goals simultaneously [136]. Although imposed selection pressures make it possible to observe evolution over time, experiments following experimental evolution have so far taken the form of snapshots after certain numbers of generations. With regard to climate change, experimental evolution allows comparing results across time points, which could provide valuable information concerning adaptive plasticity, genetic adaptation, inbreeding, adaptive divergence or co-evolutionary dynamics between interacting species.

Certainly, the classic experimental evolution approach is not suitable to study organisms with very long generation times or large home range sizes, such as large terrestrial and aquatic vertebrates. Due to many practical reasons, therefore, experimental evolution has so far been mostly applied to relatively few model systems. However, in order to improve our understanding of the evolutionary processes shaping biodiversity following climate change in nature, experimental evolution has a great potential to be expanded to many non-model organisms or even communities and to become much more methodologically sophisticated. In particular, with regard to biomass and species diversity in nature, many organisms are not considered for research, although their life span would allow experimental evolution studies. We hence would like to encourage researchers to use long-term experimental evolution on non-model organisms, novel organisms and communities. In addition, environmental conditions could be simulated more realistically in order to test impacts of climate change; for example, increasing mean temperatures or increasing extreme weather events [127]. In the following subchapters, we review experimental evolution methods and propose novel directions for future studies.

### 5.1. Selection Line Characteristics

Experimental evolution is usually based on replicated lines with no/low genetic variation or replicated lines with standing genetic variation. Classically, experimental evolution with unicellular organisms, such as *E. coli,* has used multiple identical clones to investigate evolution, with resulting variation stemming solely from novel mutations [137]. Similarly, studies with multicellular organisms such as *Drosophila melanogaster* make use of iso-female lines (*i.e.* founded with one female) to simulate clonal lines [138]. Generally, though, multicellular organisms are more frequently used in experimental evolution experiments where populations start with standing genetic variation [139]. Initial genetic variation can be manipulated to varying degrees as part of the investigation of the selection pressures under study. With regard to climate change, experimental evolution could hence be applied to investigate the evolutionary potential of populations with different initial genetic diversities qualitatively and quantitatively. 

A further climate change-relevant application of experimental evolution is to manipulate the size or density of different populations. Using this idea, it has been shown that allopatric populations of the fly *Sepsis cynipsea* have a larger potential to diverge reproductively if the populations were of high density rather than low density [140,141]. This finding has also been confirmed in another species [142]. Such an approach could be employed to obtain standardized information about the minimal population size necessary to survive different strengths of environmental change. 

Using small and rapidly replicating organisms for such experiments has the advantage that evolutionary consequences can be monitored over many generations, for example, in order to measure time to extinction, such as shown by Bell and Gonzalez [143] or Drake and Griffen [144]. Nevertheless, the same principles of manipulating effective and operational population sizes could be applied more widely, such as on organismal communities using mesocosms as proposed by Cohen *et al*. [145]. In general, to represent natural systems more realistically, replicated selection lines should be viewed as multiple populations, where different environmental treatments can be applied. By using cages in the field, such as the artificial ponds used by Bolnick [146], even organisms, which do not easily breed in the lab, could be used in future studies.

### 5.2. Evolutionary Drivers and Mechanisms

In experimental evolution experiments, it is possible to manipulate natural selection, sexual selection, drift, bottlenecks, etc., including different levels of strength and combinations of these processes [147]. Natural selection can be imposed by subjecting populations to altered environmental conditions, which has been applied in many studies investigating adaptation to, for example, different temperatures [148,149], CO_2_ levels [150], novel resources [151], starvation [152] or desiccation [153]. How climate change could be simulated within this framework is discussed below in more detail.

In selection lines it is possible to enforce different mating systems, as for example in Holland and Rice [154], or different sex ratios, such as in Michalczyk *et al*. [155], to vary sexual selection pressure. In order to change the mating system in a population, the reproducing generation is only allowed limited access to specific mates. For example, strict genetic monogamy completely removes sexual selection, and this can be achieved by randomly forming mating pairs. In contrast, polyandry and polygyny allow sexual selection to act via competition and choice mechanisms. Thus, it is possible to contrast a selection regime where sexual selection is entirely absent (monogamy) *versus* a regime where sexual selection is present (polyandry or polygyny). Using different sex ratios to start each generation is similar, but allows investigation of different sexual selection intensities rather than a presence/absence dichotomy. A female-biased sex ratio reduces sexual selection intensity (*i.e.* decreased opportunities for female choice and male-male competition), whereas a male-biased sex ratio increases sexual selection pressure. This can be utilized to approximate natural populations where various factors such as population density or sex ratio may decrease or increase sexual selection pressure.

Drift, bottlenecks and adaptability can be investigated by manipulating the initial population size and the number of individuals allowed to contribute to each generation. Starting with small populations (e.g., 100 or less individuals) greatly increases the likelihood of stochastic effects influencing evolution and potentially lowers the adaptive potential [156,157]. However, using certain model organisms (e.g., microorganisms and small arthropods such as *Drosophila* or *Tribolium*) it is also possible to investigate much larger population sizes [143]. Bottlenecks can potentially be an issue in experimental evolution, because often only a subset of individuals is allowed to contribute to the next generation, contrary to natural conditions where, in principle at least, the whole population has the chance to reproduce. Thereby, genetic diversity diminishes, generation for generation, as a side effect of the experimental set-up. Nevertheless, such mechanisms could also be deliberately promoted as part of an experimental treatment, for example, to investigate founder effects or minimum viable population sizes during climate change [156].

### 5.3. Ecological Reality

Climate change can be implemented in experimental evolution in the beginning so as to represent a sudden shift in conditions, simulating the colonization of a new habitat, a land-use change or extreme weather events [158,159]. For example, a physiological study on thermal tolerance could subject populations of a study organism to certain elevated temperatures and use experimental evolution to investigate up to which temperature the organism can adapt. Alternatively, environmental conditions can be manipulated more realistically between and within generations to simulate changing conditions as experienced by natural populations facing climate change [160,161,162]. The IPCC report [127] states specific predictions as to how environmental conditions might change over time. Therefore, future experiments could try to account for the complexity of how single environmental factors might change, and also for interactions between different factors. For example, to investigate global warming, temperatures could be raised gradually or temperatures could fluctuate with increasing mean temperatures. Contrasting these regimes could help disentangle effects of warming *per se* and effects stemming from changes in extreme temperatures. Additionally, changing conditions over time would enable manipulation of the mode of selection, such as directional *versus* diversifying, and also the strength of natural selection. A further advantage would be that selection occurs over a longer time period and, therefore, sexual reproduction can generate novel phenotypes, which might be crucial for successful adaptation to novel conditions. In addition, if experimental evolution can be performed over a very long time course, there might be the possibility to observe benefits of novel mutations.

In contrast to the classic lab setting, ecological reality could be achieved more easily in field experiments, especially if taking advantage of the naturally occurring environmental fluctuations. Small caged populations could thereby be subjected to either the natural conditions, as a control treatment, or to a manipulated treatment where environmental factors are altered. For example, effects of climate change on humidity could be studied by adding or removing water and global warming could be simulated using infrared radiators as done by Harte *et al*. [163]. 

Besides implementing abiotic factors more realistically, future experimental evolution studies could also be greatly improved by incorporating spatial aspects of climate change consequences [164]. The classic experimental evolution set-up with allopatric selection lines could be extended to a meta-population scenario by using multiple replicates of different population sizes. Migration could then be quite simply performed by exchanging individuals among populations at a given rate [165,166,167]. In particular, with regard to adaptation and speciation, gene flow and incoming migrants are expected to play important roles (see section 4). Using a meta-population experimental evolution set-up, it would be possible to investigate the consequences of climate change, habitat fragmentation and deterioration in combination. For example, populations experiencing climate change could simultaneously be subdivided over time, or habitats could be artificially impoverished by manipulating the available resources [168]. Furthermore, conservation strategies, such as artificially relocating individuals or providing dispersal corridors, could be tested. 

### 5.4. Species and Species Interactions

Although single species studies are valuable, they do not capture the full picture, as interactions between species, such as competition, can affect evolutionary responses to changing environments [169,170,171,172,173]. To accurately assess impacts of climate change on biodiversity, it would hence be valuable to apply experimental evolution approaches incorporating realistic species networks [174]. Thereby, it will be necessary to apply the method to non-model organisms and to investigate interacting and co-evolving pairs and groups of species. For studies on host-parasite co-evolution experimental evolution is already in use [175,176,177,178,179,180,181,182,183,184]. In addition, experimental evolution has successfully been established to investigate predator-prey and higher food-chains dynamics [185,186,187]. Nevertheless, there is still an underused potential for experimental evolution. As the classic experimental evolution approach is restricted to organisms reproducing in a confined setting with short generation times, organisms such as arthropods, which fulfill these prerequisites, should be utilized much more widely. With regard to the enormous species richness and abundance, more different insects could be investigated, in particular, as they are expected to respond in disparate ways to climate change [8]. In the future, however, experimental evolution should also be considered in novel areas, such as invasion biology (*i.e.* resident and incoming species interact in a disturbed environment). For example, by taking experimental evolution outdoors, this approach could be applied to many more organisms. In particular, many species, which are threatened by climate change, might be difficult to breed under lab conditions. Nevertheless, experimental evolution could be developed further in order to incorporate such organisms, which are more challenging to study in the lab. Depending on the organisms of interest, experimental evolution could be performed in multiple closed or open greenhouses, aviaries, cages, artificial or natural ponds *et cetera*.

### 5.5. Assessing Fitness

A clear advantage of experimental evolution is that responses to selection can be tracked over time. This could be extremely valuable, because it is conceivable that short-term fitness changes may differ from long-term changes. This was the case, for example, in *Callosobruchus maculatus* beetles, where the rate of adaptation to a novel food source differed between treatments with or without sexual selection [123]. This shows that taking multiple serial measurements is potentially very important. Experimental evolution should hence focus increasingly on temporal aspects of climate change, for example, by tackling the following questions: Do phenotypic and genetic adaptive responses arise sequentially or simultaneously? How fast does adaptation progress in response to different strengths of selection and based on different population characteristics? What are the dynamics of fitness changes, e.g., linear or exponential? Which reproductive traits change when over time, and are there general trends across organisms? How do different modes of environmental change (e.g., rapid or continuous) affect responses to selection over time? In addition to helping resolve these rather fundamental evolutionary questions, experimental evolution could also contribute to more applied questions, such as predicting species fitness following climate change. With regard to making conservation decisions, it might be helpful to not only theoretically model species abundance or distribution, but also to experimentally simulate certain management scenarios. Specifically, semi-natural experimental evolution experiments could be designed in order to assess short and long-term changes in fitness due to climate change. For example, regarding species extinctions due to environmental changes, researchers could assess experimentally how much biodiversity loss can be sustained by different ecosystems until productivity and nutrient-cycles are endangered. Such controlled experiments would be extremely valuable, in particular in order to identify the key species for maintaining ecosystem function.

To achieve a better overall understanding of evolutionary change, it would be crucial to standardize fitness measurements and assess multiple traits. Particularly, it may be expected that life-history strategies, such as reproduction-survival trade-offs, might shift in response to selection [114]. For example, results regarding a decrease in reproduction following a rapid change in environmental conditions might be very difficult to interpret if information about reproductive efforts later in life and longevity is missing. Therefore, we would like to emphasize once more the usefulness of measuring lifetime reproductive success at the individual level where possible, or alternatively, population growth or decline as a measure of total fitness.

## 6. Conclusions and Future Directions

A vast number of studies illustrate the very diverse immediate effects of environmental factors and hence climate change on reproduction leading to alterations on genetic, phenotypic and behavioral levels. Estimating reproductive fitness of affected populations may be crucial for predicting impacts of climate change for biodiversity; however, because of complex interactions among environmental effects, the overall reproductive output is difficult to foresee. Climate change will undoubtedly affect natural selection pressure as well as sexual selection regimes. Therefore, reproduction will be subject to a complex interplay of shifting selection pressures and this may alter adaptation, reproductive isolation and extinction risk. We urgently need an improved understanding of how natural and sexual selection shape biodiversity in detail, and how a changing environment will affect these processes.

In order to provide evolutionarily meaningful results concerning population fitness, for example to use as input data for models monitoring biodiversity or making conservation management decisions, it will be necessary to coordinate experimental methods and the traits of interest. More specifically, detailed multi-generational data on reproductive rate and longevity could be used to improve predictions regarding the minimum viable population size in a population viability analysis or the minimal habitat size to protect endangered species. Therefore, we propose making greater efforts to measure lifetime reproductive success as a common currency, as this can be used, for example, to calculate population growth rates. Furthermore, evolutionary biology provides useful resources for tracking long-term changes in reproductive output over multiple generations. In particular, we feel that experimental evolution could be used more extensively for simulating climate change, because it is an enormously versatile method. Crucially, it provides the opportunity to implement multiple environmental factors and their interactions as well as additionally manipulating selection on reproduction. In particular, there is a lack of experimental evolution studies using non-model organisms, or addressing interactions between several species or different metapopulation structures. In general, we suggest taking a step away from laboratory common garden settings towards applying more ecologically relevant environments, incorporating greater complexity and ultimately performing semi-natural experimental evolution in the field. With regard to tackling the challenges of climate change, ecologists and evolutionary biologists could hence benefit from increasingly working in tandem.
